# Immediate Cardiovascular Responses to Isometric Handgrip Exercise in Young Adults in Puri, Odisha: A Cross-Sectional Study

**DOI:** 10.7759/cureus.112083

**Published:** 2026-07-05

**Authors:** Debasish Bagh, Kailash Bhue, Nigamananda Tripathy, Devasis Naik, Sagarika Nayak, Minati Mohapatra

**Affiliations:** 1 Physiology, Shri Jagannath Medical College and Hospital, Puri, IND

**Keywords:** blood pressure, cardiovascular response, handgrip dynamometer, hypertension, isometric handgrip exercise, pulse rate

## Abstract

Background

Hypertension is a major contributor to global morbidity and mortality and is increasingly prevalent among young adults. While aerobic exercise is well established in blood pressure (BP) management, the role of isometric handgrip exercise (IHE) remains underexplored.

Objective

To evaluate the immediate effects of a single session of IHE on cardiovascular parameters such as BP and pulse rate (PR) in healthy young adults.

Methods

This analytical cross-sectional study included 87 normotensive individuals aged 21-26 years. Participants performed a standardized IHE protocol at 30% of maximal voluntary contraction. BP and PR were recorded at baseline, during, and one hour post exercise. Statistical analysis was performed using repeated measures analysis of variance and paired t-tests, with p ≤ 0.05 considered significant.

Results

Significant increases in systolic BP, diastolic BP, pulse pressure, mean arterial pressure, and PR were observed during exercise, with p-values < 0.001. After one hour, all BP parameters showed significant reductions compared to baseline. PR increased immediately post exercise and decreased after one hour, which was also found to be statistically significant with a p-value less than 0.001.

Conclusion

A single session of IHE produces transient elevations, followed by modest reductions in BP and PR. These findings suggest that IHE may serve as a simple, time-efficient, and non-pharmacological approach for cardiovascular health improvement.

## Introduction

Hypertension or high blood pressure (BP) has caused around 7.5 million deaths all over the globe, which is approximately 12.8% of global mortality [1]. Nearly 58 million disability adjusted life years (DALYS) or 4% of total DALYS have been related to hypertension [1]. As per the data from the World Health Organization, hypertension is directly responsible for about 62% of strokes and 49% of coronary artery disease throughout the world [2]. BP in the young adult population is considered to be a prediction tool for the future incidence of cardiovascular events [2]. Prevalence of hypertension among young adults has reached 15%-20%, which may be attributed to obesity and poor lifestyle [3]. Physical activity has been proven to be a beneficial intervention in managing most non-communicable diseases. As aerobic exercise training, dynamic exercise training has been shown to produce reductions in BP [4]. Isometric handgrip exercise (IHE) is a type of static resistance exercise, where muscle tension changes with constant muscle length [3]. Unlike isotonic exercises, in isometric exercise, only small groups of muscles remain in a contracted state, causing compression of blood vessels and reduction of perfusion to the active muscle [5]. In recent times, with the advent of digital platforms, people have gone through a sedentary routine, which leads to chronic morbidities, including coronary artery disease and hypertension [6,7]. Literature on the effects of isometric exercise on cardiovascular parameters is limited, particularly in the eastern part of Odisha. With this background, the present study was planned to study the pattern of immediate changes of certain cardiovascular parameters like systolic BP (SBP), diastolic BP (DBP), mean arterial pressure (MAP), pulse pressure (PP), and pulse rate (PR) or heart rate (HR) after a "standard protocol of single bout isometric exercise" in a population of young adults from the eastern part of Odisha, India.

## Materials and methods

Study design and setting

An analytical cross-sectional study was conducted at Shri Jagannath Medical College and Hospital, Puri. The study was carried out over a duration of one month (between February and March 2026) by Bachelor of Medicine and Bachelor of Surgery (phase III, part 2) students during their elective posting in the Department of Physiology, under the guidance of senior faculty members.

Participant selection

Sample Size Calculation

To determine the minimum sample size required for this study, a power analysis was conducted based on a predicted correlation coefficient (r) of 0.35. With the significance level (alpha) set at 0.05 and a desired statistical power of 80% (1-beta = 0.80), the calculation utilized Fisher’s z-transformation to normalize the correlation distribution. Under these parameters, the minimum required sample size was determined to be 62 participants. To maintain the same buffer for potential attrition or incomplete data as in the study done by Shruthi et al. (approximately 39%), the final enrolment target was adjusted to 87 participants [8].

A total of 87 (100%) young adults, with no hypertension or stage 1 hypertension, aged 21-26 years, were recruited from patient attendants at the hospital using a consecutive sampling method. The young, healthy attendants of the patients attending the tertiary care hospital in Puri were expected to represent the healthy young community of Puri town. Ethical approval for the study was granted by the Institutional Ethics Committee (IEC) of Shri Jagannath Medical College and Hospital, Puri. All participants provided written informed consent before the commencement of the study.

Inclusion Criteria

Healthy individuals with no hypertension or stage 1 hypertension, those not yet started on pharmacotherapy, from both male and female genders, were included with BP less than 139/89 mmHg. Stages of hypertension were determined according to the 2025 American Heart Association (AHA)/American College of Cardiology (ACC) guidelines [5].

Exclusion Criteria

Known cases of hypertension (stage 2 and above) and cases receiving pharmacotherapy for hypertension, diabetes, cardiovascular, respiratory, or neurological disorders were excluded. Participants with recent medical or surgical illness and musculoskeletal conditions affecting the wrist or hand were not included in the study. Individuals with symptoms such as chest pain, dizziness, or syncope during exertion were also excluded from this study.

Exercise protocol

IHE was performed using a handgrip spring dynamometer. The maximum voluntary contraction (MVC) and its percentages were performed in a sitting posture with the dominant hand. The position of the elbow was maintained at 90 degrees with the arm parallel to the body. The subjects performed three MVCs sustained for five seconds, with five minutes of recovery between them. The MVC value was determined as the highest value obtained from the three measurements with less than 10% of variation between them. Following a three-minute rest period, subjects performed sustained contractions at 30% of their MVC for two minutes using their dominant hand [4].

Data collection

Blood Pressure Measurement Validation

To ensure the clinical validity and reliability of the data, BP measurements were conducted in strict accordance with the 2025 AHA/ACC hypertension guidelines. All BP data were collected using an automated, digital monitor (Omron Healthcare, Kyoto, Japan) that has been independently validated according to the Association for the Advancement of Medical Instrumentation (AAMI)/European Society of Hypertension (ESH)/International Organization for Standardization (ISO) 81060-2:2018 protocol [5]. Participants were seated in a quiet, temperature-controlled environment for at least five minutes prior to the first measurement. The following procedural standards were maintained to minimize measurement bias.

To ensure participant readiness, they were advised to have an empty bladder and abstain from caffeine, nicotine, and strenuous exercise for at least 30 minutes preceding the session. To maintain proper posture and positioning, subjects were seated with their back supported, feet flat on the floor, and legs uncrossed. The arm was supported on a flat surface at the level of the right atrium (heart level). Participants were stratified by body mass index (BMI) to account for its known correlation with systemic vascular resistance [5]. To avoid the risk of cuff size hypertension, an appropriately sized cuff, determined by mid-upper arm circumference, was applied directly to the skin. Proper cuff placement was verified to ensure the bladder encircled at least 80% of the arm circumference. A quiet environment was ensured to avoid the alerting or stress response. Participants remained silent, and no electronic devices like phones or digital watches were permitted during the measurement process [5].

BP and PR were measured using an Omron digital BP and pulse apparatus (Omron Healthcare). The different BP parameters were SBP, DBP, PP (SBP minus DBP), and MAP (DBP plus one-third of PP). Measurements were recorded at three specific time points: before starting the IHE (baseline), during the performance of the exercise, and one hour after the completion of the exercise. The IHE was performed on the dominant hand, and the BP and PR were checked on the other hand.

Statistical analysis

The collected data were expressed as mean ± standard deviation (SD). Repeated measures analysis of variance (RM ANOVA) and paired t-tests were utilized to compare the physiological values recorded before, during, and after the exercise protocol. Mean differences and confidence intervals of each parameter were determined. Statistical significance was defined as a p-value ≤ 0.05. All statistical analyses were performed using SPSS version 20.0 (IBM Corp., Armonk, NY) and Microsoft Excel (Microsoft Corporation, Redmond, WA).

## Results

A total of 87 (100%) young adults were enrolled from both genders, which included 57 (65.5%) males and 30 (34.5%) females, with a mean age of 23.36 ± 1.75 years (Table 1).

**Table 1:** Demographic description of the study participants. SD: standard deviation. P < 0.05: Statistically significant using an independent t-test for age comparison between genders.

Characteristic	Total (N = 87)	Male (n = 57)	Female (n = 30)	p-value
Age (years), Mean ± SD	23.36 ± 1.75	23.86 ± 1.85	22.40 ± 1.04	<0.001
Range (min - max)	21 - 26	21 - 26	21 - 24	-
Gender, n (%)				
Male	57 (65.5%)			
Female	30 (34.5%)			

A repeated measures ANOVA was conducted to evaluate the effect of IHE on cardiovascular parameters across three time points: before, during, and one hour after IHE. The mean values of all parameters increased during the isometric exercise from the baseline and again decreased one hour post exercise. There was a statistically significant effect of time on SBP, as evident from the F-values and p-values. A similar significant effect of time was observed on DBP, PR, PP, and MAP (Table 2).

**Table 2:** Repeated measures ANOVA (within-subject effects) for different study parameters. IHE: isometric handgrip exercise; SBP: systolic blood pressure; DBP: diastolic blood pressure; PP: pulse pressure; MAP: mean arterial pressure; mmHg: millimeters of mercury; bpm: beats per minute; df: degrees of freedom. P < 0.05: Statistically significant, derived from repeated measures ANOVA test.

Variables	Baseline (Mean ± SD)	During IHE (Mean ± SD)	1-hour post IHE (Mean ± SD)	F-value (df = 2,172)	p-value
SBP (mmHg)	125.37±7.60	146.98±5.86	115.95±6.23	3971.54	<0.001
DBP (mmHg)	80.39±6.06	92.94±4.12	72.02±6.69	673.64	<0.001
PP (mmHg)	44.98±3.10	54.03±3.67	43.93±4.93	187.86	<0.001
MAP (mmHg)	95.38±6.45	110.95±4.45	86.67±6.11	1603.02	<0.001
Pulse rate (bpm)	79.79±8.06	103.08±8.04	70.99±4.65	2325.93	<0.001

A post hoc analysis was performed to compare the variables at three different timelines: baseline versus during IHE, during IHE versus one hour post IHE, and baseline versus one hour post IHE. During the performance of IHE, all cardiovascular parameters demonstrated a robust and statistically significant increase compared to baseline levels, with highly positive t-values and p-values less than 0.05 for all parameters. SBP showed the most substantial acute increase, with the 95% confidence interval (CI) indicating a consistent rise across the cohort. MAP and DBP followed a similar upward trajectory, while the effect on PP was minimal. Additionally, PR exhibited a significant increase during the exercise bout (Table 3).

**Table 3:** Post hoc analysis of all variables: baseline vs. during IHE, during vs. one hour post IHE, and baseline vs. one hour post IHE. IHE: isometric handgrip exercise; SBP: systolic blood pressure; DBP: diastolic blood pressure; PP: pulse pressure; MAP: mean arterial pressure; mmHg: millimeters of mercury; bpm: beats per minute; CI: confidence interval. P < 0.05: Statistically significant, derived from paired t-test.

Variables	Change: Baseline vs. During IHE, Mean difference (95% CI)	t-value	p-value	Change: During vs. one hour post IHE, Mean difference (95% CI)	t-value	p-value	Change: Baseline vs. one hour post IHE, Mean difference (95% CI)	t- value	p-value
SBP (mmHg)	+21.61 (20.88, 22.34)	58.74	<0.001	−31.02 (−31.84, −30.21)	−75.48	<0.001	−9.41 (−9.97, −8.86)	−33.72	<0.001
DBP (mmHg)	+12.55 (11.50, 13.60)	23.72	<0.001	−20.92 (−22.4, −19.44)	−28.17	<0.001	−8.37 (−9.15, −7.58)	−21.19	<0.001
PP (mmHg)	+9.06 (8.08, 10.03)	18.43	<0.001	−10.10 (−11.64, −8.57)	−13.11	<0.001	−1.05 (−1.82, −0.27)	−2.69	0.008
MAP (mmHg)	+15.57 (14.73, 16.41)	36.89	<0.001	−24.29 (−25.36, −23.21)	−44.94	<0.001	−8.72 (−9.33, −8.10)	−28.07	<0.001
Pulse (bpm)	+23.29 (22.46, 24.11)	56.04	<0.001	−32.09 (−32.97, −31.21)	−72.62	<0.001	−8.80 (−9.97, −7.64)	−15.07	<0.001

At the one-hour post-exercise recording, significant reductions in the measurements were observed. The parameters not only decreased from the "during IHE" values but also showed a reduction from the baseline measurements. The mean differences for both SBP and DBP at one hour post exercise were negative, with CIs that did not cross zero, reinforcing the reliability of this BP reduction. MAP and PR also remained significantly lower than baseline. While PP showed a statistically significant reduction, the magnitude of the mean difference was smaller compared to the other BP parameters (Table 3).

## Discussion

The present study investigated the acute and immediate cardiovascular responses to IHE among a cohort of 87 young, healthy participants with no or stage 1 hypertension (Table 1). Our findings demonstrate a dual-phase hemodynamic response: a significant acute increase in BP and PR during exercise, followed by a substantial reduction in these parameters one hour post IHE (Table 2). These results are in coherence with the growing evidence describing isometric exercise as a potent intervention for cardiovascular modulation.

Mechanisms of the acute pressor response

The significant increase in SBP, DBP, and MAP during the 30% MVC may be due to the exercise pressor response. This response is primarily driven by sustained muscle contraction, which increases total peripheral resistance and sympathetic activity [7]. Almost similar findings were obtained in a recent study done by Dutta et al. in 2023 [7]. The substantial increase in PR observed during the exercise phase may be suggestive of withdrawal of parasympathetic tone and an increase in sympathetic outflow. Interestingly, the significant rise in PP during IHE may be a reflection of an increase in stroke volume and arterial stiffness during the period of sustained contraction [7].

Post-exercise recovery and hypotensive effect

The most notable finding of this study is the significant reduction in SBP and DBP after one hour of IHE, with values dropping below the initial baseline. This phenomenon, known as post-exercise hypotension, is hypothesized to result from a persistent decrease in total peripheral resistance that is not fully compensated by changes in cardiac output [8]. The mean reductions of approximately 9.41 mmHg in SBP and 8.37 mmHg in DBP observed in our study are of clinical interest. Shruthi et al. found similar results in their recently published study [8]. The consistent negative mean differences and narrow confidence intervals in our data reinforce the reliability of IHE in inducing this hypotensive state.

The mechanisms underlying these effects may include reduced sympathetic outflow, improved endothelial function, and enhanced vasodilation responses [8]. Additionally, autonomic modulation with increased parasympathetic activity may contribute to post-exercise recovery [7].

The significant rise in PR during IHE reflects increased sympathetic nervous system activity and elevated cardiac output in response to sustained muscle contraction [7]. The subsequent decline in PR after one hour, falling below the baseline levels, suggests enhanced parasympathetic reactivation and autonomic recovery [9]. This biphasic response highlights the dynamic cardiovascular adaptation to isometric stress and supports the role of autonomic regulation in post-exercise recovery [8].

Clinical implications for lifestyle medicine

Current hypertension management guidelines are increasingly emphasizing non-pharmacological interventions. As this study followed a cross-sectional design with a small sample size, the results cannot be generalized. But it paves the path to future research on the use of IHE as a time-efficient and accessible tool for BP regulation among pre-hypertensives and patients with stage 1 hypertension. Unlike dynamic aerobic exercise, IHE requires minimal equipment and can be performed by individuals with limited mobility, making it a versatile option in lifestyle medicine [10]. This procedure may prove to be a part of lifestyle modification.

Limitations

Despite the above findings, the study has limitations. A relatively small sample size reduces the generalizability of the findings, and the absence of a control group to avoid confounders is a limitation. It focuses only on short-term outcomes among pre and stage 1 hypertensives; hence, the long-term effects of IHE and the variations among stage 2 and above hypertensives cannot be outlined.

## Conclusions

The study concludes that IHE may appear to be a practical and efficient intervention that requires minimal time and resources. A single session can induce transient but potentially beneficial changes in cardiovascular parameters among normal individuals and pre and stage 1 hypertensives.

Given its simplicity and cost-effectiveness, IHE may serve as a useful adjunct in cardiovascular risk reduction strategies. Further research may establish a dose-response relationship between the amount of isometric exercise and the reduction in baseline BP and PR. This underscores the ability of the study outcome in favor of clinical translation. However, further studies involving larger populations, prospective study designs, long-term interventions, hypertensive subjects of higher stages, and hypertension with other comorbidities are necessary to establish its clinical application.

## References

[1] Polo-López A, Calatayud J, Palau P, López-Bueno L, Núñez-Cortés R, Andersen LL, López-Bueno R (2024). Joint associations of handgrip strength and physical activity with incident cardiovascular disease and overall mortality in the UK Biobank. Clin Nutr.

[2] Celis-Morales CA, Welsh P, Lyall DM (2018). Associations of grip strength with cardiovascular, respiratory, and cancer outcomes and all cause mortality: prospective cohort study of half a million UK Biobank participants. BMJ.

[3] World Health Organization (2002). The World Health Report: 2002: Reducing Risks, Promoting Healthy Life. https://iris.who.int/server/api/core/bitstreams/a47f00a6-4b58-4e53-b1ce-ed71d2256208/content.

[4] Gois MO, Simões RP, Porta A, Kunz VC, Pastre CM, Catai AM (2020). Cardiovascular responses to low-intensity isometric handgrip exercise in coronary artery disease: effects of posture. Braz J Phys Ther.

[5] Jones DW, Ferdinand KC, Taler SJ (2025). 2025 AHA/ACC/AANP/AAPA/ABC/ACCP/ACPM/AGS/AMA/ASPC/NMA/PCNA/SGIM guideline for the prevention, detection, evaluation and management of high blood pressure in adults: a report of the American College of Cardiology/American Heart Association Joint Committee on Clinical Practice Guidelines. Hypertension.

[6] Barbosa RM, Dos Santos AC, do Sacramento MS, Dos Santos CP, Souza PE, Santana US, Petto J (2025). Effect of isometric resistance exercise on blood pressure in normotensive adults: a systematic review of randomized clinical trials. Ann Transl Med.

[7] Dutta A, Bhowmik A (2023). Acute effects of single-bout isometric handgrip exercise on selected cardiovascular parameters in young normotensive adults at a tertiary care center of West Bengal. Ann Med Sci Res.

[8] Shruthi BR, Jadhav V, Tyagi A, Srivastava S (2025). Association between handgrip strength and cardiovascular parameters in healthy individuals. Eur J Cardiovasc Med.

[9] Zhang F, Luo B, Bai Y, Zhang Y, Huang L, Lu W (2024). Association of handgrip strength and risk of cardiovascular disease: a population-based cohort study. Aging Clin Exp Res.

[10] Quattrocchi A, Garufi G, Gugliandolo G, De Marchis C, Collufio D, Cardali SM, Donato N (2024). Handgrip strength in health applications: a review of the measurement methodologies and influencing factors. Sensors (Basel).

